# Frequency cluster formation and slow oscillations in neural populations with plasticity

**DOI:** 10.1371/journal.pone.0225094

**Published:** 2019-11-14

**Authors:** Vera Röhr, Rico Berner, Ewandson L. Lameu, Oleksandr V. Popovych, Serhiy Yanchuk

**Affiliations:** 1 Neurotechnology Group, Technische Universität Berlin, Berlin, Germany; 2 Institut für Theoretische Physik, Technische Universität Berlin, Berlin, Germany; 3 Institut für Mathematik, Technische Universität Berlin, Berlin, Germany; 4 National Institute for Space Research (INPE), São José dos Campos, São Paulo, Brazil; 5 Institut für Physik, Humboldt-Universität zu Berlin, Berlin, Germany; 6 Institute of Neuroscience and Medicine - Brain & Behaviour (INM-7), Research Centre Jülich, Jülich, Germany; 7 Institute for Systems Neuroscience - Medical Faculty, Heinrich-Heine University Düsseldorf, Düsseldorf, Germany; Georgia State University, UNITED STATES

## Abstract

We report the phenomenon of frequency clustering in a network of Hodgkin-Huxley neurons with spike timing-dependent plasticity. The clustering leads to a splitting of a neural population into a few groups synchronized at different frequencies. In this regime, the amplitude of the mean field undergoes low-frequency modulations, which may contribute to the mechanism of the emergence of slow oscillations of neural activity observed in spectral power of local field potentials or electroencephalographic signals at high frequencies. In addition to numerical simulations of such multi-clusters, we investigate the mechanisms of the observed phenomena using the simplest case of two clusters. In particular, we propose a phenomenological model which describes the dynamics of two clusters taking into account the adaptation of coupling weights. We also determine the set of plasticity functions (update rules), which lead to multi-clustering.

## Introduction

Clustering of the dynamics and coupling is observed at several scales of the brain structure and function. For example, in the data measured by the functional magnetic resonance imaging (fMRI), the brain networks form functional clusters that can be seen in the matrices of the functional and effective connectivities for task-based and task-free (resting state) paradigms [1–6]. Clustering has also been observed for dynamic functional connectivity, where the time courses of the connectivity exhibit a few discrete states with well pronounced clusters [7]. Disruption of such clustered states of connectivity may be associated with some brain disorders [8, 9]. It is therefore important to investigate the emergence of clustering in neural populations that we address in this study.

Neural networks are able to adapt their structure depending on the activity of the nodes or external stimuli [10]. One of the possible mechanisms of such an adaptation, which may lead to persistent changes in neural connections and relate to learning and memory, is synaptic plasticity [11]. The efficacy of synapses to transmit the electrical potential between neurons may increase or decrease depending on the mutual neural activity, which results in short- or long-term potentiation or, respectively, depression of synapses [12, 13]. An example is spike timing-dependent plasticity (STDP) which describes the synaptic weight change as a function of the difference of spiking times between pre- and post-synaptic neurons [14–18].

One of the famous plasticity rules, the Hebbian rule, assumes that the modifications of the synaptic weights are driven by correlations in the firing activity of pre- and post-synaptic neurons. More specifically, it assumes that those connections are potentiated, for which one neuron contributes to the firing of another [11]. Nevertheless, in many publications, the Hebbian rule is considered in a more narrow sense of a closeness between the spiking times: the smaller the distances between the spikes are the higher is the potentiation of the corresponding synapse [19, 20]. In this work, we are dealing with spike-based learning rules rather than rate-based.

Previous studies of neural networks with STDP showed that such networks can evolve and create various coupling structures. For instance, the weights can exhibit stable localized spatial structures, that can be interpreted as receptive fields [21]. These structures can be either unidirectionally of bidirectionally coupled, depending on the plasticity rule or external input properties. The STDP mechanism plays an important role in temporal coding of information by spikes [14, 21]. On the one hand, a synchronized firing in neural ensembles with STDP can be stabilized through potentiation of synaptic coupling by stimulation-induced transient synchronization of neurons [22–25]. On the other hand, a desynchronized state can lead to a depression of synaptic weights [22, 23]. Thus, neural networks with plasticity are prone to a co-existence of different stable dynamical and structural states, which may be realized by choosing appropriate initial conditions or stimulation procedures.

Human brain networks demonstrate different degrees of modularity, sometimes with hierarchical features [26–30]. Recently, a hierarchical clustering was observed in phenomenological models of adaptive networks of phase oscillators [31–33]. As a result of an adaptation, the network evolved into groups of strongly-connected clusters, while the coupling between the groups was depressed. The stability analysis of such clusters reveals [33, 34] that the preferred stable cluster configuration corresponds to significantly different sizes of the clusters. The dynamics within each cluster are frequency-synchronized, while the frequencies between clusters differ. Thus, self-organized emergence of clusters leads to the emergence of different collective frequencies in the system. The multi-stability of such clusters was also observed in ensembles of Morris-Lecar bursting neurons with STDP in [35].

In this paper we report on the phenomenon of clustering with respect to connectivity and frequencies in a network of adaptively coupled Hodgkin-Huxley (HH) neurons. The spike timing-dependent adaptation is considered to be symmetric as experimentally found for hippocampal synapses [36] and can also be derived from asymmetric STDP for an “effective time window” [37]. Then the observed clusters are bidirectionally coupled [35]. Splitting of a neural population to a few clusters synchronized at different frequencies could lead to a slow waxing and waning of the amplitude of the mean field, where the clusters transiently gather together and move apart as the time evolves [35]. The frequency of such a modulation of the mean neural activity could be much smaller than the firing rate of individual neurons and depends on the differences between the clusters’ frequencies. The emergence of synchronized clusters could explain the origin of the low-frequency modulation of the spectral power of macroscopic brain signals like local field potentials (LFP) or electroencephalographic (EEG) signals in higher frequency bands, which also correlates with slow oscillations of the blood oxygen level-dependent (BOLD) signal measured by fMRI [38–41]. Several other modeling studies have also reported on clustering in the neural populations with plasticity [35, 42, 43]. These clusters have been observed for different models that ranged from simple phase oscillators to the models of spiking and bursting neurons and demonstrate stability with respect to heterogeneity of the interacting neurons and random perturbations [35, 42, 43]. In this paper we provide a simple phenomenological model and explain a mechanism governed by synaptic plasticity of the stabilization of such clusters in a neural population.

The structure of the paper is as follows. In the first section we present the model. The next section shows numerically observed multi-clusters. The detailed mechanisms of the stability of frequency clusters is explained afterwards using the simplest case of two clusters. Then we propose a phenomenological model, which describes the dynamics of two clusters taking into account the adaptation of the weights. The model is shown to reflect not only qualitative, but also some basic quantitative properties of the two-cluster formation. We also determine the set of plasticity functions (update rules), which lead to the clustering.

## Materials and methods

### Model

The network of *N* HH neurons is described by the following system [24, 25, 44, 45]

$$\begin{array}{ll} C{\dot{V}}_{i} & =\;I_{i}-g_{Na}m_{i}^{3}h_{i}(V_{i}-E_{Na})-g_{k}n_{i}^{4}(V_{i}-E_{K})-g_{L}(V_{i}-E_{L})-\frac{\left(V_{i}-E_{r}\right)}{N}\sum_{j=1}^{N}\kappa_{ij}s_{j},\\ {\dot{m}}_{i} & =\;\alpha_{m}(V_{i})(1-m_{i})-\beta_{m}(V_{i})m_{i},\\ {\dot{h}}_{i} & =\;\alpha_{h}(V_{i})(1-h_{i})-\beta_{h}(V_{i})h_{i},\\ {\dot{n}}_{i} & =\;\alpha_{n}(V_{i})(1-n_{i})-\beta_{n}(V_{i})n_{i},\\ {\dot{s}}_{i} & =\;\frac{5(1-s_{i})}{1+e^{\left(\frac{-V_{i}+3}{8}\right)}}-s_{i}. \end{array}$$ (1)

Here *V*_*i*_ is the potential of the *i*-th neuron with the corresponding equilibrium potentials *E*_*Na*_ = 50mV, *E*_*K*_ = −77mV, and *E*_*l*_ = −54.4mV. *C* = 1*μF*/*cm*^2^. Our choice of *E*_*r*_ = 20mV corresponds to the excitatory neurons. *m*, *h*, and *n* are gating variables, and their dynamics depend on opening and closing rates

$$\begin{array}{ll} \alpha_{m}\left(V\right) & =\frac{0.1V+4}{1-e^{\left(-0.1V-4\right)}},\\ \beta_{m}\left(V\right) & =4e^{\left(\frac{-V-65}{18}\right)},\\ \alpha_{h}\left(V\right) & =0.07e^{\left(\frac{-V-65}{20}\right)},\\ \beta_{h}\left(V\right) & =\frac{1}{1+e^{\left(-0.2V-3.5\right)}},\\ \alpha_{n}\left(V\right) & =\frac{0.01V+0.55}{1-e^{\left(-0.1V-5.5\right)}},\\ \beta_{n}\left(V\right) & =0.125e^{\left(\frac{-V-65}{80}\right)}. \end{array}$$

The parameters are *g*_*Na*_ = 120mS/cm^2^, *g*_*K*_ = 36mS/cm^2^, and *g*_*l*_ = 0.3mS/cm^2^. The constant current *I*_*i*_ is set to 9*μA*/*cm*^2^ so that the individual neurons are identical and oscillatory.

The synaptic input current from *j*-th neuron is scaled by the synaptic strength *κ*_*ij*_, which changes due to plasticity. The adaptation of *κ*_*ij*_ occurs discontinuously whenever one of the neurons *i* or *j* spikes. More specifically, the discontinuous change is given by the following plasticity function

$$\begin{array}{c} \kappa_{ij}\to \{\begin{array}{cc} 0, & \;\text{if}\;\kappa_{ij}+\delta W\left(\Delta t_{ij}\right)<0\\ \kappa_{ij}+\delta W\left(\Delta t_{ij}\right), & \;\text{if}\;0\leq \kappa_{ij}+\delta W\left(\Delta t_{ij}\right)\leq \kappa_{\text{max}}\\ \kappa_{\text{max}}, & \;\text{if}\;\kappa_{ij}+\delta W\left(\Delta t_{ij}\right)>\kappa_{\text{max}} \end{array} \end{array}$$ (2)

where Δ*t*_*ij*_ = *t*_*i*_ − *t*_*j*_ is the spike time difference between the postsynaptic and presynaptic neurons; *δ* > 0 is a small parameter determining the size of the single update; *κ*_max_ > 0 is the maximal coupling; and the plasticity function [14–16, 21] is

$$\begin{array}{c} W\left(\Delta t_{ij}\right)=c_{p}e^{-\frac{|\Delta t_{ij}|}{\tau_{p}}}-c_{d}e^{-\frac{|\Delta t_{ij}|}{\tau_{d}}} \end{array}$$ (3)

with positive parameters *c*_*p*_, *τ*_*p*_, *c*_*d*_, and *τ*_*d*_. We also assume no autapses and set *κ*_*ii*_ = 0.

**Fig 1:**
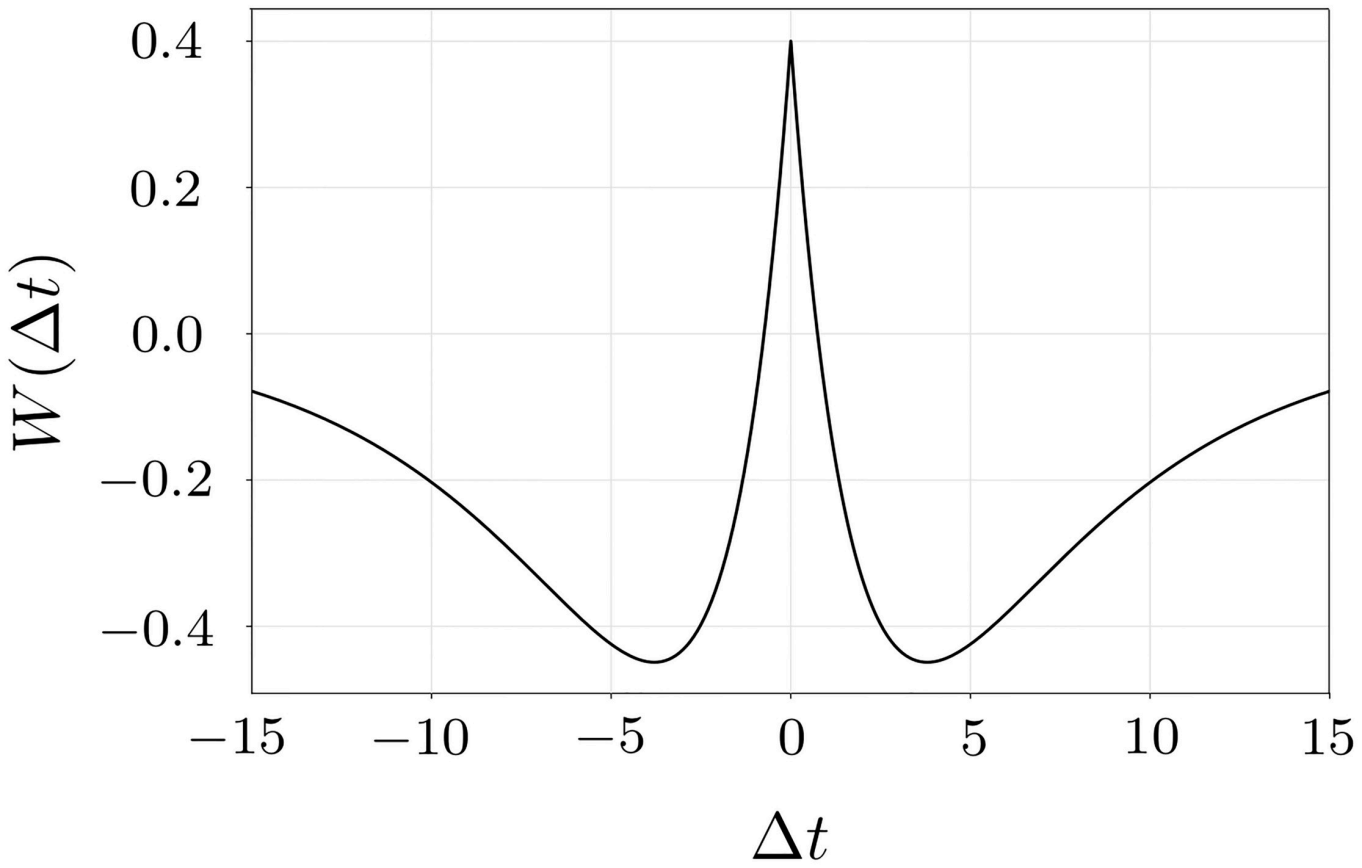
Plasticity function *W*(Δ*t*_*ij*_) for *τ*_*p*_ = 2, *τ*_*d*_ = 5, *c*_*p*_ = 2, *c*_*d*_ = 1.6.

Example of the considered plasticity function *W* used in our simulations is shown in Fig 1. This is a symmetric function, which corresponds to a potentiation of the coupling weights of the neurons with highly correlated firing. As we will discuss at the end of the results section, there is a family of plasticity functions of similar form that allow for the frequency clustering.

## Results

### Numerical observation of synchrony and frequency clustering

**Fig 2:**
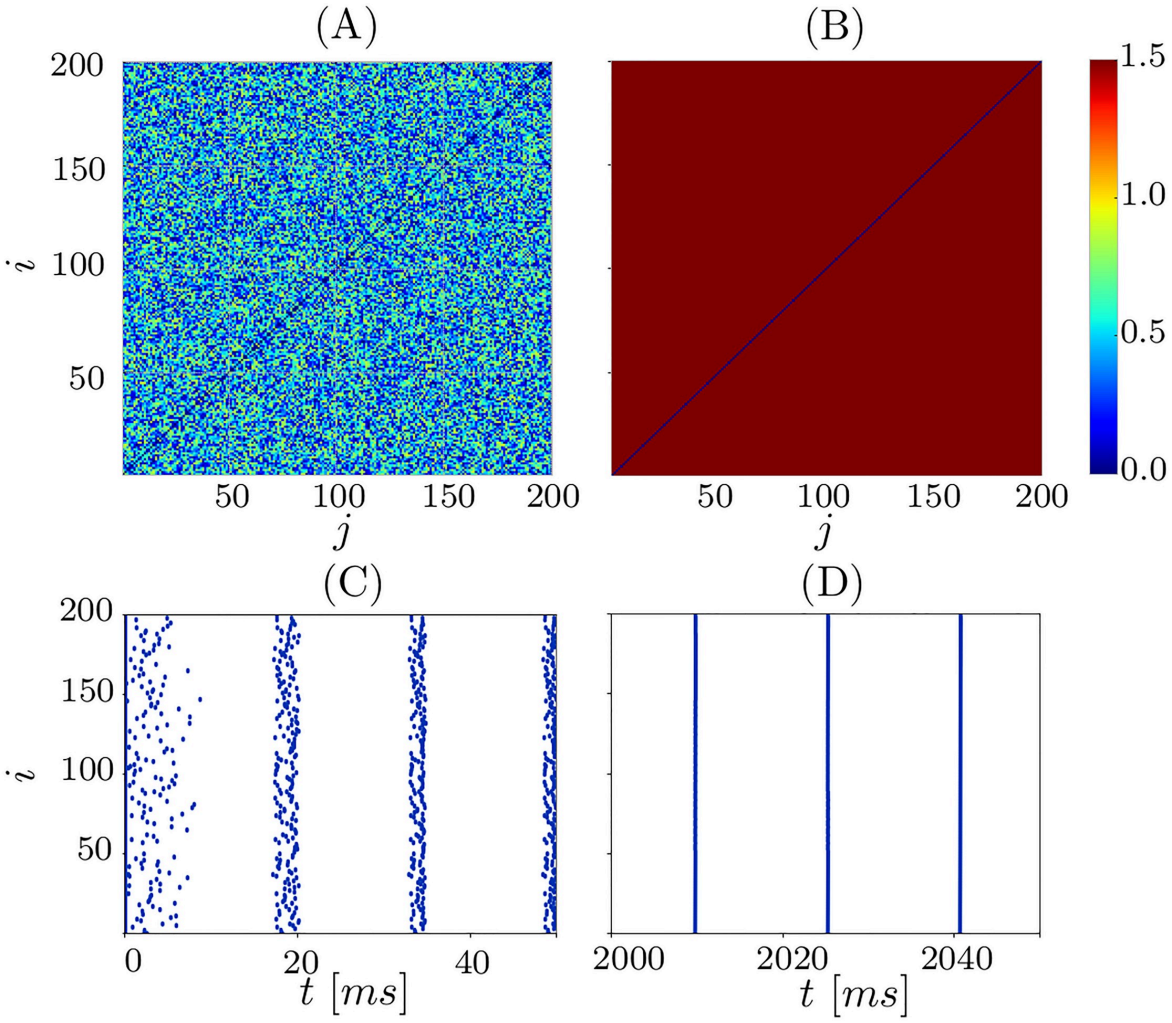
Synchronization into one Cluster. Evolution of the coupling matrix *κ*_*ij*_(*t*) starting from random initial conditions and converging to a completely synchronous state. Panel (A) shows initial coupling matrix, (B) the coupling matrix after the transient *t* = 2000ms. Raster plot of spiking times at the beginning of simulations (C) and after the transient (D). The asymptotic state (B,D) is a completely synchronized spiking with all coupling weights *κ*_*ij*_ potentiated to *k*_max_. Other parameters *N* = 200, *τ*_*p*_ = 2, *τ*_*d*_ = 5, *c*_*p*_ = 2, *c*_*d*_ = 1.6, and *κ*_max_ = 1.5.

**Fig 3:**
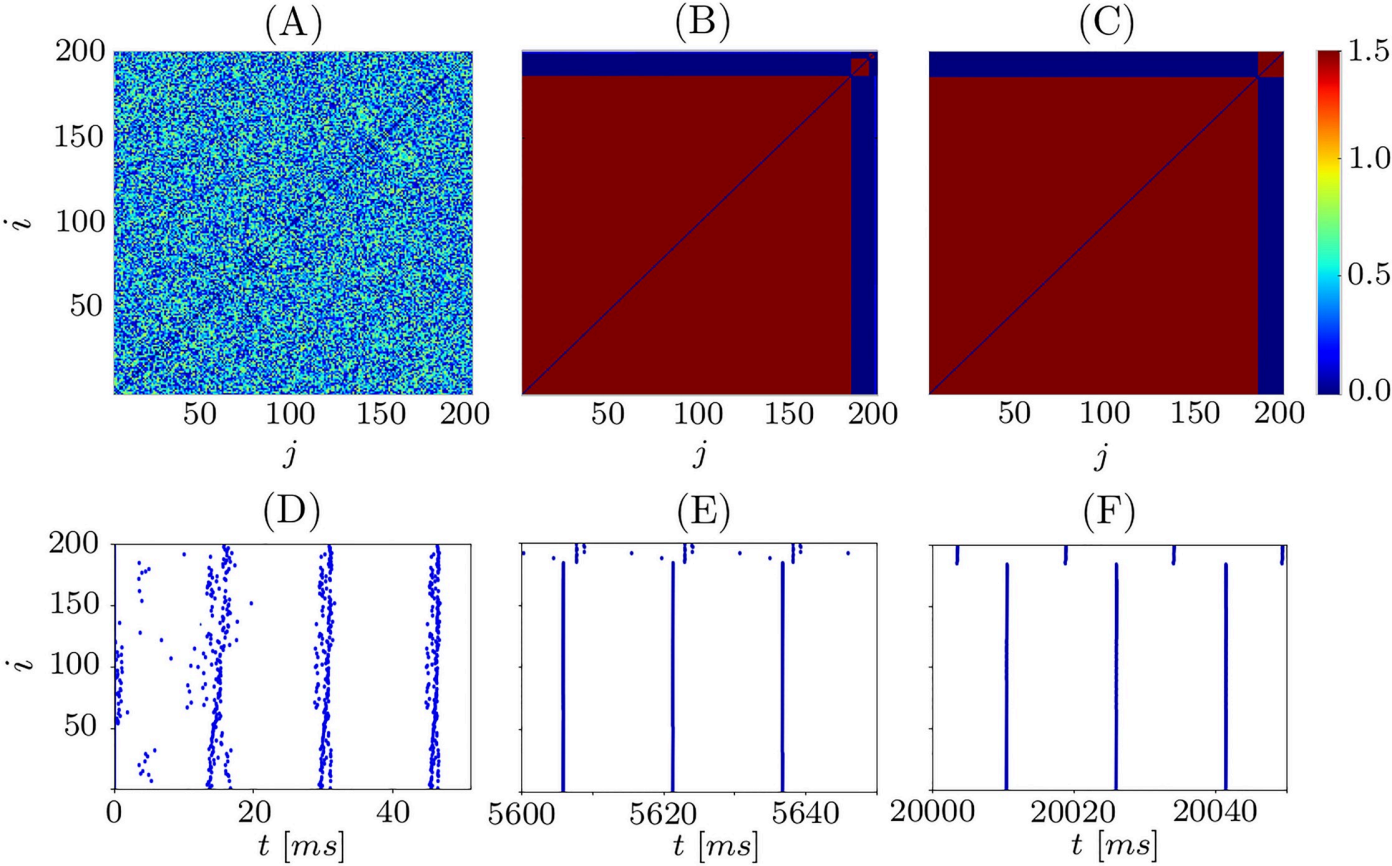
Frequency clusters. Evolution of the coupling matrix *κ*_*ij*_(*t*) starting from random initial conditions and converging to frequency clusters hierarchical in size. Panel (A) shows initial coupling matrix, (B) the coupling matrix after the transient *t* = 5600ms, and (C) *t* = 20000ms. (B-F) Corresponding raster plots of spike times. The asymptotic state (C,F) is a hierarchical cluster state with the coupling weights *κ*_*ij*_ potentiated to *k*_max_ within each cluster and small or zero otherwise. Other parameters as in Fig 2. The oscillators are ordered accordingly to their mean frequency.

In order to investigate the dynamics of network (1), we initialize the neurons and the coupling randomly and integrate the system numerically. For the parameter values *τ*_*p*_ = 2, *τ*_*d*_ = 5, *c*_*p*_ = 2, *c*_*d*_ = 1.6, and *κ*_max_ = 1.5 we observe two phenomena: complete synchronization and the emergence of frequency clusters hierarchical in size, see Figs 2 and 3, respectively.

Fig 2(A) shows the initial coupling weights, and Fig 2(C) illustrates the spike times of the neurons at the beginning of the simulation. One can observe that while the neurons start with an incoherent spiking, they enhance the coherence already after a few spikes due to the interaction between them as well as the plasticity. The plasticity potentiates the connections of neurons that fire together. A complete synchronization is established and the coupling weights increase to *κ*_max_ after a transient (Fig 2(B) and 2(D)). In a completely synchronized state, the individual neurons spike simultaneously, hence, the spike time differences Δ*t*_*ij*_ = 0.

The emergence of frequency clusters is shown in Fig 3 for two clusters. The system in Fig 3 possesses the same parameters as in Fig 2, and the difference is just another realization of random initial conditions. In contrast to the synchronized state, the final state shown in Fig 3(F) consist of two groups of synchronized neurons. These cluster states also manifest themselves as two groups of strongly coupled elements in the coupling matrix *κ* (Fig 3(C)). The coupling weights between the neurons from the different groups is very small or zero.

**Fig 4:**
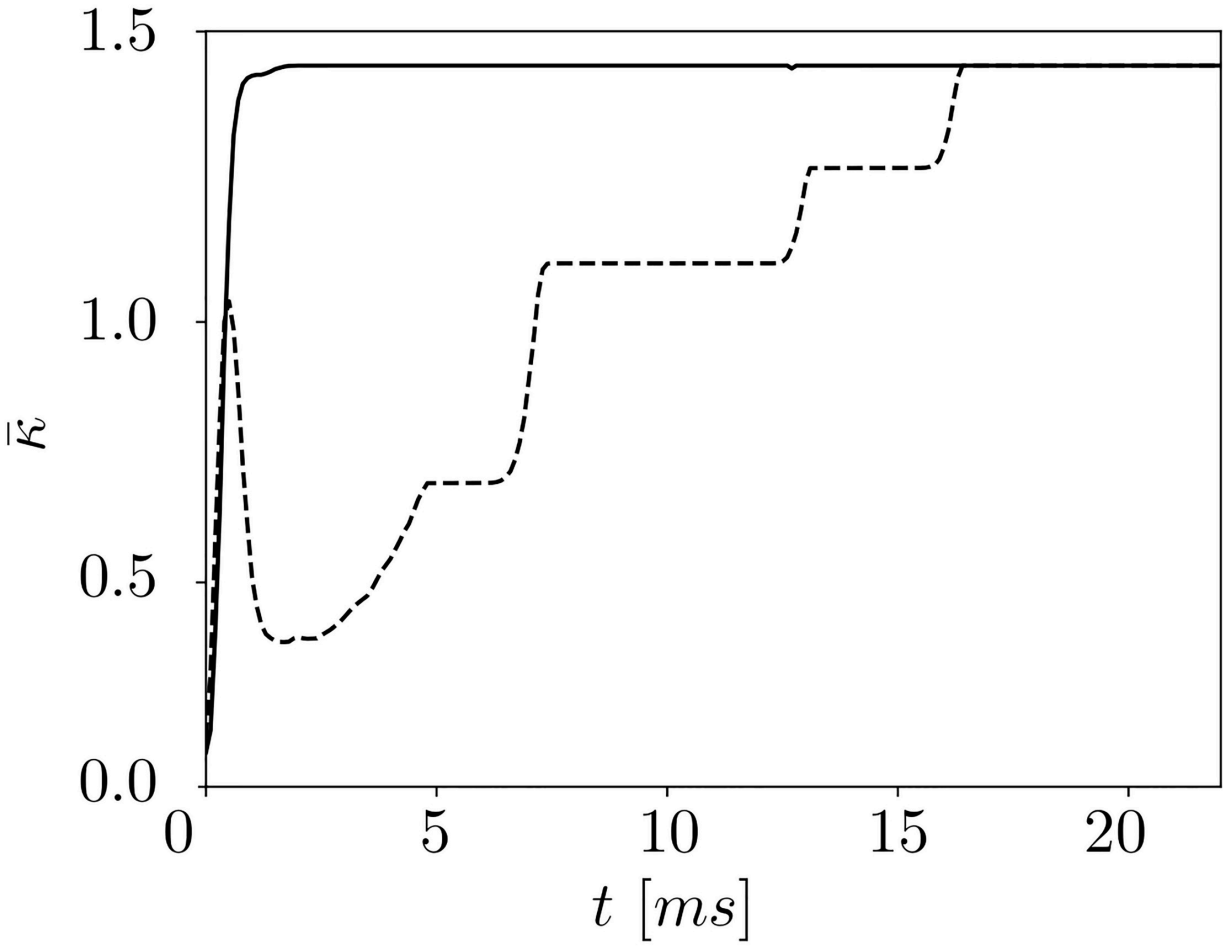
Cluster formation. Formation of individual clusters over time (corresponds to the dynamical scenario in Fig 3). The dashed and solid curves depict the time course of the mean coupling within the small and big clusters, respectively.

We observe that the largest cluster is formed rather quickly as time evolves, whereas the formation of the small cluster takes much more time. This is illustrated in Fig 4, where the time courses of the mean coupling within each of the two clusters are shown. The average coupling within the big cluster reaches its maximum fast (at *t* ≈ 1000, solid curve in Fig 4), whereas the smaller cluster in Fig 3(C) and 3(F) is formed through the merging of transient clusters and finally establishes at *t* ≈ 17000 (dashed curve in Fig 4).

**Fig 5:**
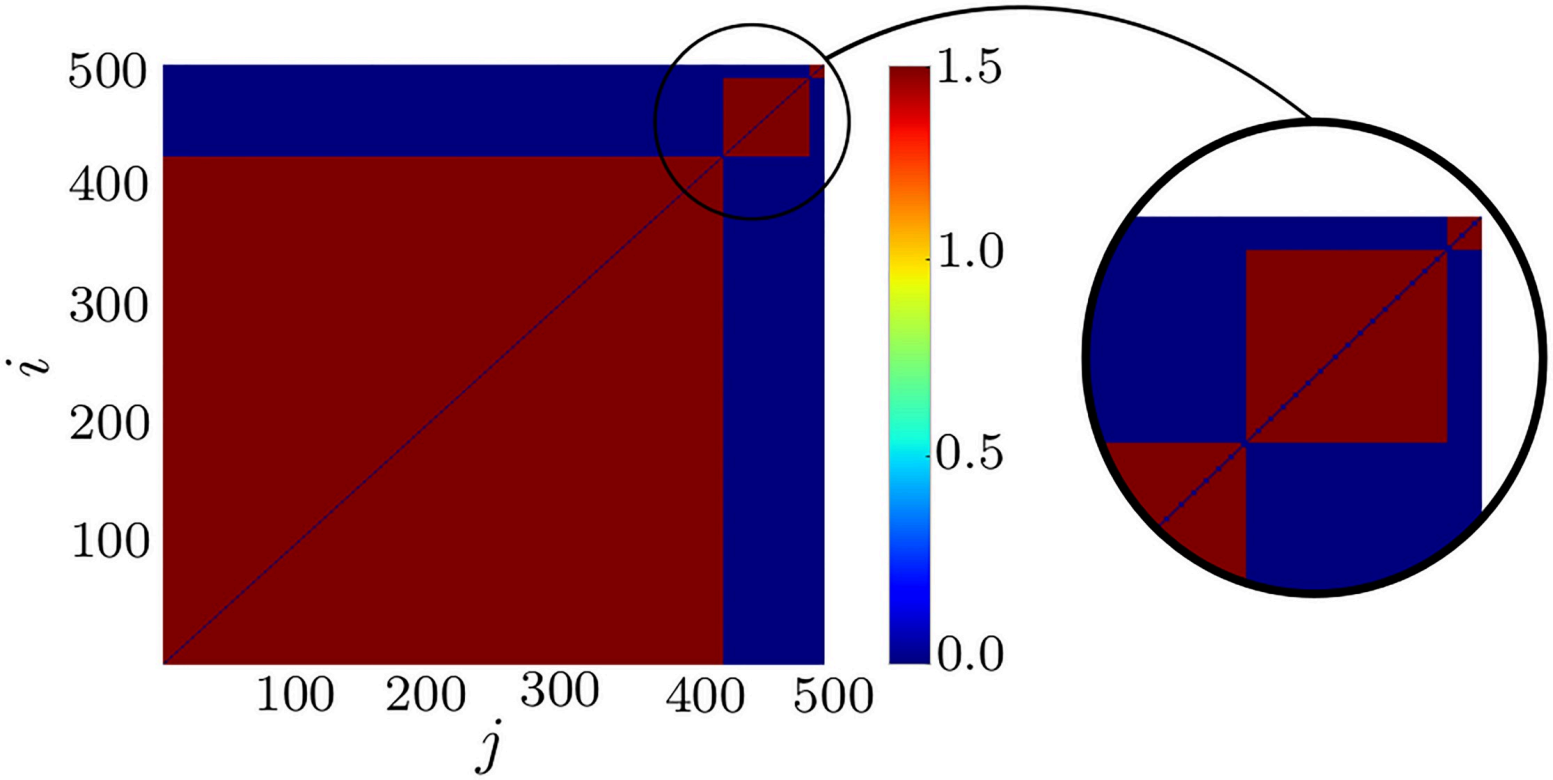
Three-cluster state. Example of a three-cluster state for *N* = 500, *τ*_*p*_ = 2, *τ*_*d*_ = 5, *c*_*p*_ = 2, *c*_*d*_ = 1.6, and *κ*_max_ = 1.5 with a random initial distribution of *κ*_*ij*_ in [0, 0.75].

For the states with more clusters, each new formed cluster is significantly smaller than the previous one, see Fig 5, where three clusters are shown. The spiking period of the cluster appears to be proportional to its size: the bigger the cluster the larger is the period. Simulation of the cases with even more clusters becomes computationally expensive due to large transients.

#### Clustering with independent random input

To investigate the robustness of our findings, we added an *α*-train as additional independent random input to the membrane potential *V*_*i*_ of every neuron:

$$\begin{array}{c} I_{i}^{input}\left(t\right)=I\left(V_{r}-V_{i}\left(t\right)\right)\sum_{\tau_{i,k}<t}\alpha \left(t-\tau_{i,k}\right)e^{-\alpha (t-\tau_{i,k})}) \end{array}$$ (4)

The Eq (4) models a postsynaptic potential (PSP) that is received by the neuron at certain random times *τ*_*i*,*k*_. The inter-spike interval is Gaussian distributed $\tau_{i,k+1}-\tau_{i,k}=\Delta \tau_{i,k}∼N\left(14ms,4ms\right)$. *α* is set to 24/〈Δ*τ*_*i*,*k*_〉.

**Fig 6:**
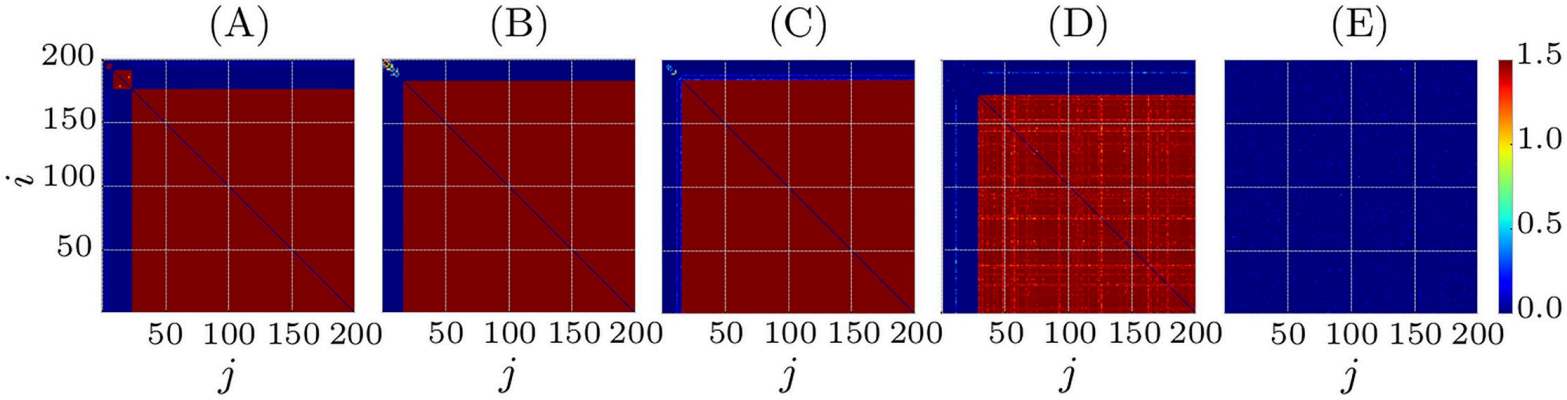
Influence of independent random input on clusters. Coupling matrices for *t* = 10000ms and different amplitudes of independent random input *I* (see Eq (4)). (A) *I* = 0.005, (B) *I* = 0.01, (C) *I* = 0.02, (D) *I* = 0.05 and (E) I = 0.07. All other parameters as in Fig 5.

The numerical simulations Fig 6 show that the clustering is still observed under the influence of random input $I_{i}^{input}\left(t\right)$ of intensity *I*. More specifically, for sufficiently weak perturbations with *I* < 0.01, all three clusters survive (Fig 6(A)). With increasing the amplitude *I*, the clusters start to decouple. The smaller clusters are affected first (Fig 6(B)–6(D)), they start desynchronizing at *I* = 0.01. The biggest cluster keeps shrinking in size while *I* is increased and finally for *I* = 0.07 the whole network decouples (Fig 6(E)).

### Two clusters in more detail

In this section we numerically show that depending on the relative size of the two clusters, such two-cluster states can be either dynamically stable or transient leading to complete synchronization. In order to investigate the cluster stability, we initialize the system in a two cluster state with the number of neurons *N*_*s*_ in the small cluster and *N*_*b*_ = *N* − *N*_*s*_ in the big cluster. The total number of neurons is set to *N* = 50. The inter-cluster couplings are set to zero initially while the intra-cluster couplings equals *κ*_max_. All neurons in the same cluster are initialized with the same initial conditions, so the clusters are fully synchronized at *t* = 0.

**Fig 7:**
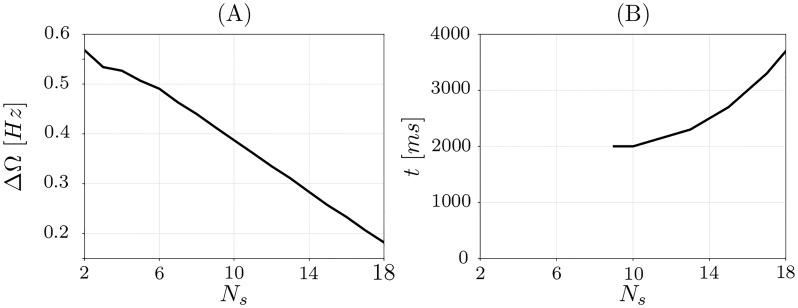
Cluster frequencies and time until fusion. (A)Difference between synchronization frequencies of the two clusters for different size of the smaller cluster *N*_*s*_. (B) Time until cluster fusion for different initial size of the smaller cluster *N*_*s*_.

**Fig 8:**
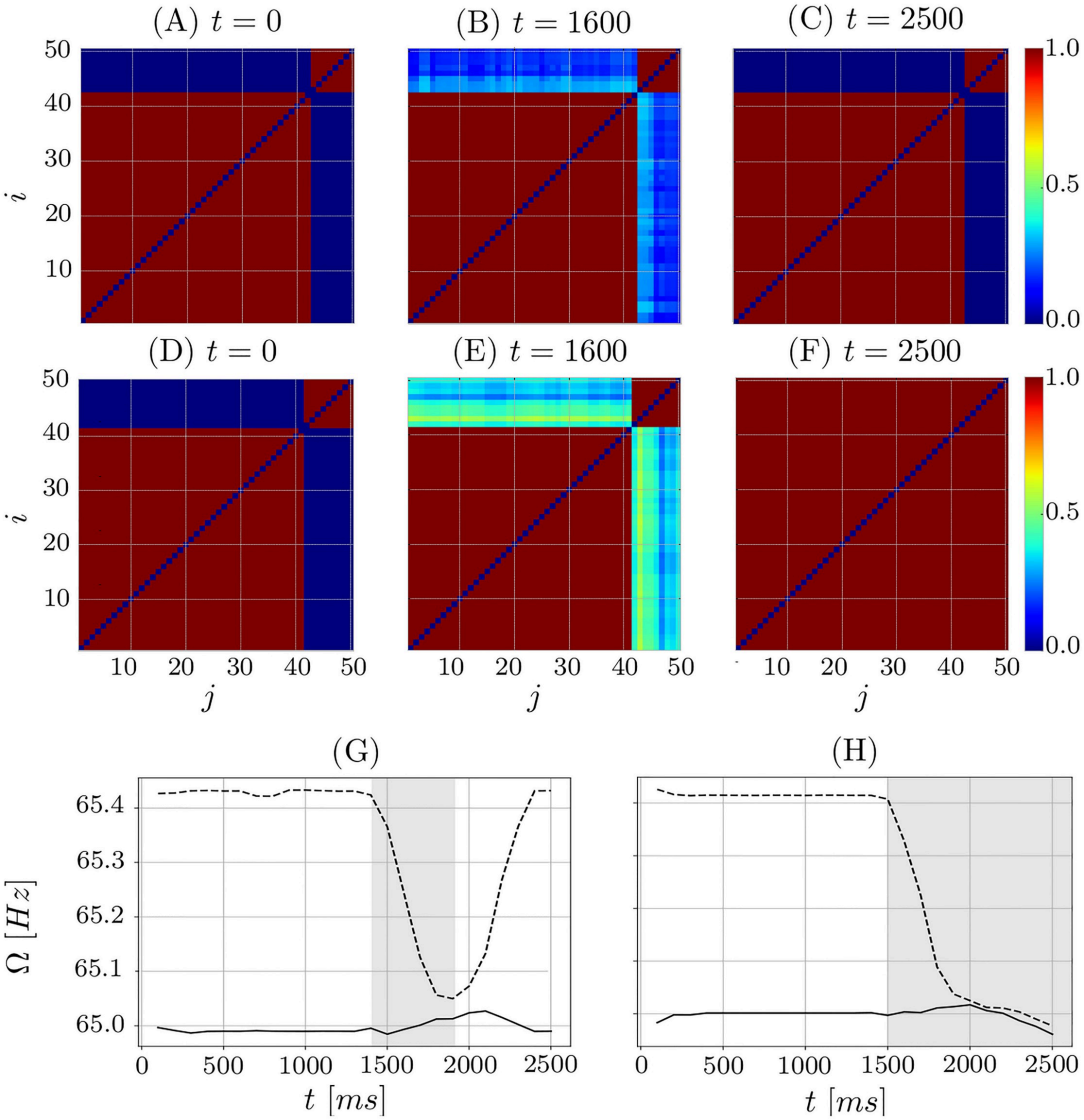
Two cases: Fusion and stable clusters. Evolution of the coupling matrix for *N* = 50 and the number of neurons *N*_*s*_ = 8 (A)-(C) and *N*_*s*_ = 9 (D)-(F) in the small cluster. In panels (A)-(C) the clusters are stable, while in (D)-(F) they are merging to one synchronous cluster. (G, H) Time courses of the spiking synchronization frequencies of small (*N*_*s*_ neurons) and large (*N*_*b*_ neurons) clusters depicted by dashed and solid curves, respectively, for (G) *N*_*s*_ = 8 and *N*_*b*_ = 42 and (H) *N*_*s*_ = 9 and *N*_*b*_ = 41. Parameter *κ*_*max*_ = 1.0.

Fig 7(A) shows frequency difference of two uncoupled clusters as a function of the size of the small cluster. The frequency difference demonstrates an almost linear dependence on the cluster size and decays as the size of the smaller cluster increases. Moreover, we also observe that clusters with sufficiently different sizes are stable while the clusters of similar sizes, in the considered case with *N*_*s*_ > 8, are transient, merge into a single cluster and eventually lead to a stable completely synchronous state, see Fig 8.

Although the threshold of how different the clusters should be in order to be stable is certainly model dependent, the synchronization of similar clusters is a general property. The merging of two clusters can be explained qualitatively as follows. Initially uncoupled clusters evolve each with their natural frequencies Ω_*s*_ and Ω_*b*_. If their sizes are different, then Ω_*s*_ ≠ Ω_*b*_, and the clusters arrive in-phase periodically with the ‘beating’ frequency ΔΩ = Ω_*b*_ − Ω_*s*_. As soon as such an in-phase episode occurs, the interspike intervals Δ*t*_*ij*_ between any two neurons from different clusters become small and, hence, due to the plasticity rule *W* (see Eq (2) and Fig 1) the inter-cluster coupling weights increase. Moreover, the duration of such an in-phase episode depends on the frequency difference between the clusters. As a result, for a small frequency difference ΔΩ, the time interval where the clusters are practically in-phase is sufficiently long in order to potentiate the coupling weights to their maximum value. This unites the two clusters into one. In contrast, for large ΔΩ, such an episode is short, and the inter-cluster coupling remains small, which keeps the clusters oscillating at different frequencies in a stable manner.

Fig 8(A)–8(C) displays an example of two-cluster stable state with *N*_*s*_ = 8. Starting from the two-cluster state, after *t* = 1500ms, the coupling between the clusters increases, see Fig 8(B) due to the “in-phase episode” when the clusters are synchronous. Afterwards, however, the inter-cluster coupling weights return to their initial configuration (Fig 8(C)), since the spike time differences for neurons from different clusters are again far enough apart to cause the depression of the inter-cluster synapses. Such a process is repeated every time the clusters meet and is typical for the stable cluster states. A typical case of transient clusters is presented in Fig 8(D)–8(F) for *N*_*s*_ = 9. The inter-cluster coupling is again potentiated when the clusters meet, but it does not decrease again, and the clusters merge in a single cluster of a fully coupled and synchronized regime (Fig 8(F)). The transient time that could be elapsed until the cluster fusion depends on the cluster size as illustrated in Fig 7(B).

Fig 8(G) and 8(H) show how the spiking frequency of the clusters change over time. During the in-phase episode, the cluster with the higher natural spiking rate slows down significantly, while the slower cluster (with larger number of neurons *N*_*b*_) speeds up a little. For a stable cluster state the cluster frequencies again deviate from each other (Fig 8(G)), whereas all neurons fire with the same frequency when the clusters unite into one (Fig 8(H)). We found this phenomenon for different numbers of neurons and different *κ*_max_. Increasing *κ*_max_ increases the initial period difference, but the behavior in general stays the same.

**Fig 9:**
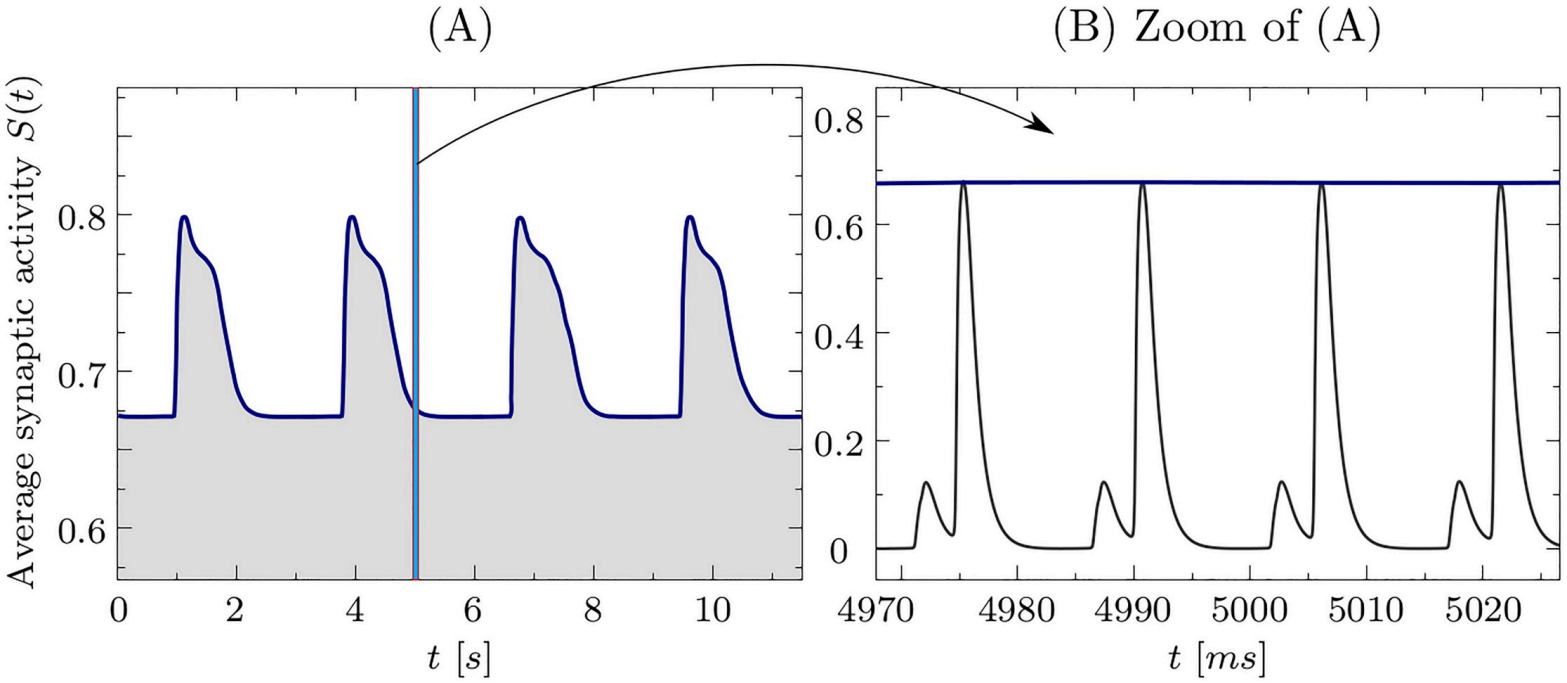
Mean synaptic activity. Mean synaptic activity *S*(*t*) of the neural population in the case of stable two cluster state. Panel (A) shows the dynamics of *S*(*t*) on the time interval of 12 s, where modulation of the amplitude (blue line) is visible, while the fast oscillations are not recognized on this timescale. The maximum amplitude corresponds to the two clusters being synchronised, while the low amplitude corresponds to the clusters being out of phase. Panel (B) shows the zoom of a small time interval. The modulation takes place on the timescale which is two orders of magnitude larger than the individual spikes of *S*(*t*) as well as individual neural spikes in both clusters. Cluster frequencies *ω*_1_ = 0.065012 kHz and *ω*_2_ = 0.065416 kHz. The corresponding period of modulation is *T* ≈ 2.5*s*.

Fig 9 shows the dynamics of the mean synaptic activity $S\left(t\right)=\frac{1}{N}\sum_{i=1}^{N}s_{i}\left(t\right)$ of the network in the case of two stable clusters, which models the dynamics of LFP. During the in-phase episodes of the two clusters, *S*(*t*) has a higher amplitude, because both clusters spike synchronously. The maximum amplitude is generated by maximum synchronization in the network. The low amplitude of *S*(*t*), on the other hand, corresponds to the time intervals when the clusters are out of phase. In the latter case, the mean synaptic activity shows two peaks, the higher peak is generated by the larger cluster and the lower by the smaller one, see Fig 9(B). For the considered case, the synchronized oscillations of individual neurons in the clusters take place at a time scale of several milliseconds (period ∼15 ms, Fig 9(B)), see also Fig 8(G) and 8(H). The neurons are tonically spiking. The frequency difference ΔΩ between clusters is, however, of the order of sub-Hz, because the corresponding cluster frequencies are close to each other (Fig 8(G) and 8(H)). Then the modulation of *S*(*t*) is observed at a much slower timescale of a few seconds, which is of two orders of magnitude slower than the intrinsic neural firing, see Fig 9(A), as observed in empirical data of the brain activity [40, 41].

In the following section, a phenomenological model is introduced in order to further investigate the dynamics of two clusters.

### Phenomenological model

#### Model derivation

In this section we introduce a reduced qualitative model for the coupling and phase difference of two clusters. The model is based on the assumption that oscillators are synchronized identically within each cluster and the coupling between the clusters is weak. As a result, the interaction between oscillatory clusters can be described in the framework of two coupled phase oscillators that are interacting via their phase differences [46–49]

$${\dot{\varphi }}_{1}\;=\;\omega_{1}-F_{1}\left(\varphi_{1}-\varphi_{2}\right),$$ (5)

$${\dot{\varphi }}_{2}\;=\;\omega_{2}-F_{2}\left(\varphi_{2}-\varphi_{1}\right),$$ (6)

where *ω*_1_ and *ω*_2_ are the natural frequencies of the individual clusters, *F*_1_ and *F*_2_ are effective interaction functions. For the phase difference *φ* = *φ*_1_ − *φ*_2_, this system reads

$$\begin{array}{c} \dot{\varphi }=\omega -F\left(\varphi \right) \end{array}$$ (7)

where *ω* = *ω*_1_ − *ω*_2_ is the difference of the natural frequencies, and *F*(*φ*) = *F*_1_(*φ*) − *F*_2_(−*φ*).

Since the clusters are synchronized for a sufficiently small frequency mismatch *ω*, the periodic interaction function *F*(*φ*) must satisfy *F*(0) = 0 and *F*′(0) > 0. The latter means that there is a stable equilibrium *φ* = 0 for small *ω*. Aiming at a qualitative insight, we further simplify the model by assuming that *F*(*φ*) = *σ* sin(*φ* + *α*), where sin *φ* can be viewed as a first Fourier harmonic of the interaction function and *σ* as an effective coupling weight. The parameter *α* = sin^−1^(*ω*/*σ*_max_) is a constant phase shift assuring that the phase difference of the synchronized cluster is zero. In fact, for small *ω*, this parameter is also small and it does not play important role in the qualitative behavior of the model apart from a small shift of the synchronized state to *φ* = 0.

Another component of the model is the plasticity-driven changes of the coupling *σ*. In order to derive the equation for *σ*, we consider the STDP update in the case of a periodic motion of the clusters. We assume that the coupling *σ* is proportional to an averaged coupling between the clusters. This is a natural assumption in the case of weakly coupled systems. Let us find out how the update of the intercluster coupling depends on the phase difference *φ*. For a given phase difference *φ* and the frequencies $\omega_{1}=\bar{\omega }+\omega /2$, $\omega_{2}=\bar{\omega }-\omega /2$ (here we introduced the mean frequency $\bar{\omega }$), the spiking period of the both clusters can be approximated as $T\approx 2\pi /\bar{\omega }$ up to small terms of order *ω*, and the distance Δ*T* between the spikes of two clusters

$$\begin{array}{c} \Delta T=[T\frac{\varphi_{1}}{2\pi }-T\frac{\varphi_{2}}{2\pi }]\;\text{mod}\;T=[T\frac{\varphi }{2\pi }]\;\text{mod}\;T\approx \frac{\varphi \;\text{mod}\;2\pi }{\bar{\omega }}. \end{array}$$

**Fig 10:**
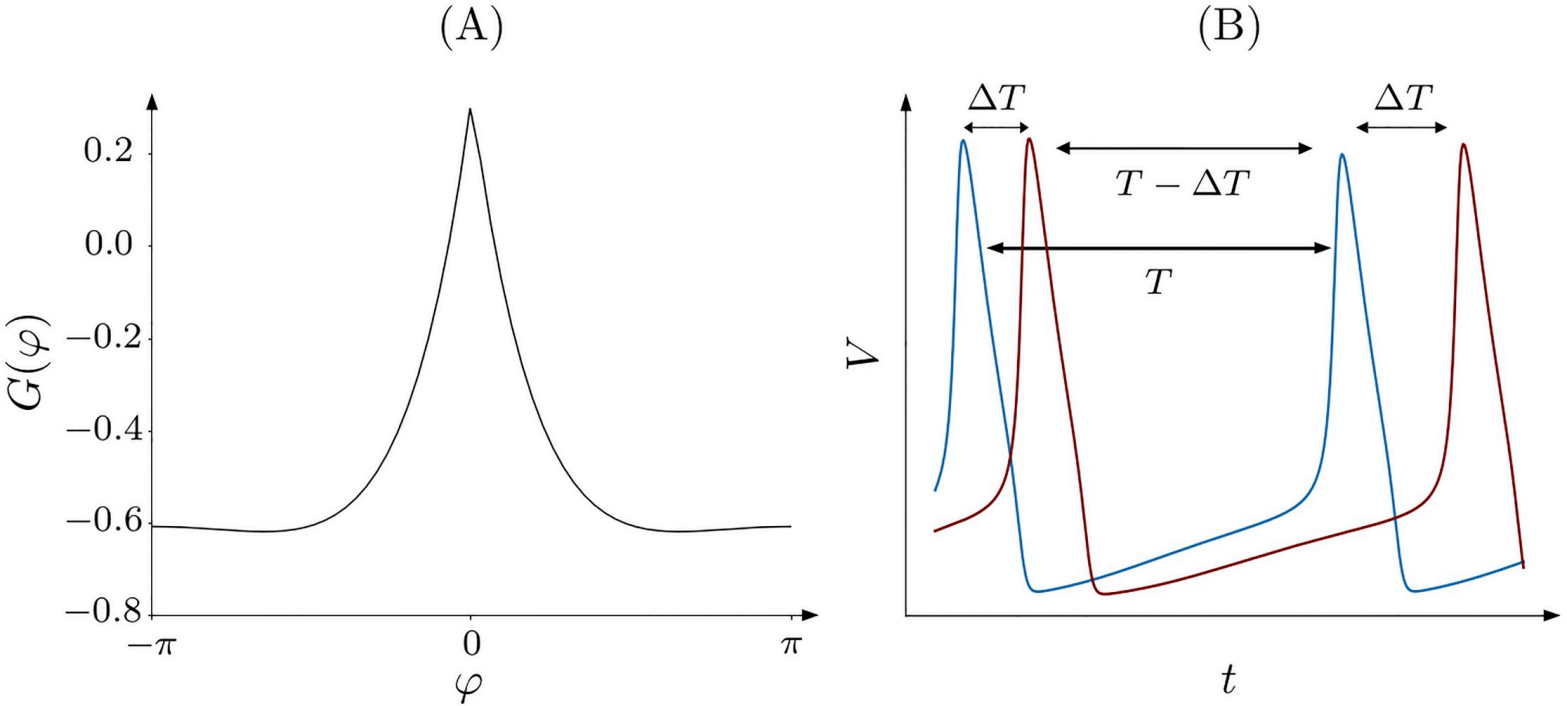
Update function G. (A) Update function *G*(*φ*) for *τ*_*p*_ = 2, *τ*_*d*_ = 5, *c*_*p*_ = 2, and *c*_*d*_ = 1.6. (B) Schematic spiking of two oscillators with spike time difference Δ*T* and periods close to *T*.

Since the spike time differences Δ*T* and *T* − Δ*T* occur recursively, see Fig 10(B), the updates per unit time sum to the function

$$\begin{array}{c} \frac{\delta }{T}\left(W\left(T-\Delta T\right)+W\left(\Delta T\right)\right)=\frac{\delta \bar{\omega }}{2\pi }G\left(\varphi \right), \end{array}$$ (8)

where

$$\begin{array}{c} G\left(\varphi \right)≔W(\frac{2\pi -(\varphi \;\text{mod}\;2\pi )}{\bar{\omega }})+W(\frac{\varphi \;\text{mod}\;2\pi }{\bar{\omega }}). \end{array}$$ (9)

Since the update of *σ* is proportional to the obtained function, and taking into account the smallness of *δ*, this update can be written as $\dot{\sigma }=\varepsilon G\left(\varphi \right)$, where *ε* is a small parameter of the coupling adaptation that controls the scale separation between the fast dynamics of the clusters and the slow dynamics of the coupling.

Additionally, the coupling strength *σ*(*t*) should be bounded to the interval [0, *σ*_max_] by imposing cut-off conditions. More specifically, the derivative $\dot{\sigma }$ is discontinuous at the boundaries *σ* = 0 and *σ* = *σ*_max_, i.e. $\dot{\sigma }=\max\left\{0,\varepsilon G\left(\varphi \right)\right\}$ for *σ* = 0 and $\dot{\sigma }=\min\left\{0,\varepsilon G\left(\varphi \right)\right\}$ for *σ* = *σ*_max_. The considered cut-off corresponds to “hard” bound conditions [50]. Another possibility would be “soft” or “multiplicative” bounds [51], when the update is proportional to the distance to the boundary. We consider here the hard bound, since it corresponds to the hard bound of the STDP rule for HH system.

The final phenomenological model reads as follows

$$\dot{\varphi }\;=\;\omega -\sigma \text{sin}\left(\varphi +\alpha \right),$$ (10)

$$\dot{\sigma }\;=\;\varepsilon \;\cdot \;\{\begin{array}{ll} G(\varphi ) & \text{for}\;0\;<\;\sigma \;<\;\sigma_{\text{max}},\\ \text{max}\{0,\;G(\varphi )\} & \text{for}\;\sigma =\;0,\\ \text{min}\{0,\;G(\varphi )\} & \text{for}\;\sigma =\;\sigma_{\text{max}}. \end{array}$$ (11)

with frequency mismatch *ω* > 0 and *α* = sin^−1^(*ω*/*σ*_max_).

#### Properties of the model

**Fig 11:**
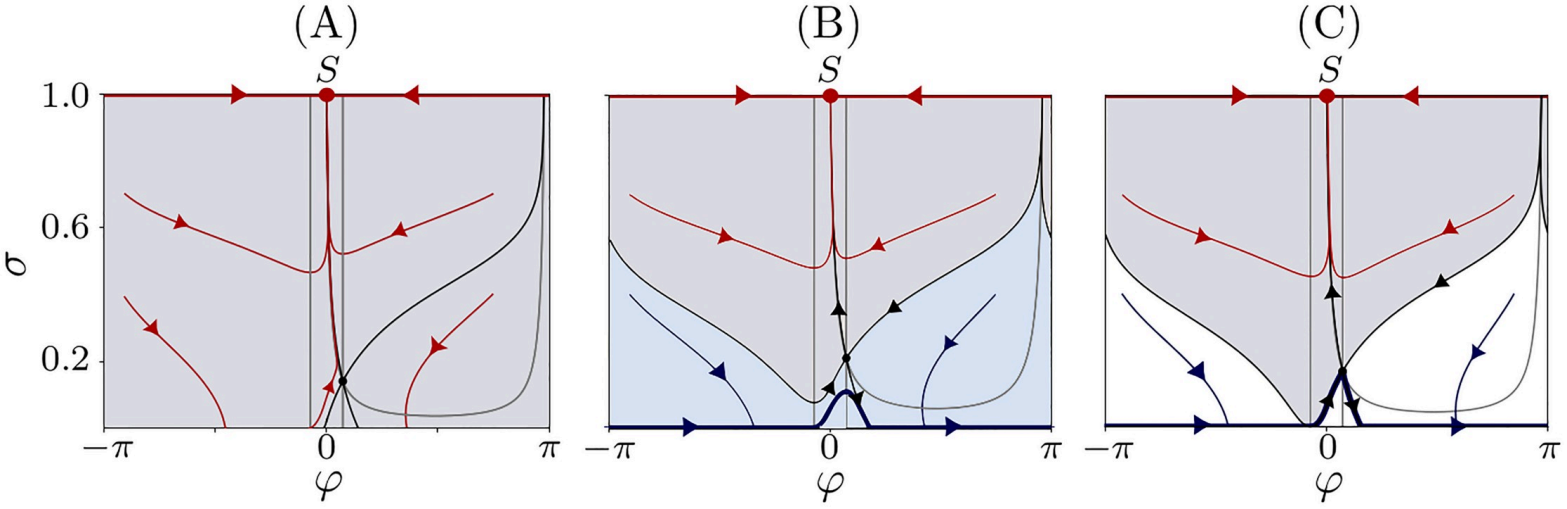
Phase portrait phenomenological model. Phase portraits of model (10)–(11) for (A) monostable regime of complete synchronization; (B) co-existence of stable synchronized and clustered states; and (C) bifurcation moment of transition between the phase portraits illustrated in (A) and (B). The basins of attraction of the synchronized regime (point *S*), clustered state (limit cycle indicated by thick black curve) and the saddle fixed point (φ*, σ*) are depicted by gray, blue, and white colors, respectively. The nullclines of the system and stable and unstable manifolds of the saddle point are indicted by the thin gray and black curves, respectively. Parameters (A) *ω* = 0.037 kHz, (B) *ω* = 0.06 kHz, (C) *ω* ≈ 0.455 Hz, and the other parameters *τ*_*p*_ = 2, *τ*_*d*_ = 5, *c*_*p*_ = 2, *c*_*d*_ = 1.6, and *ε* = 0.08.

Phase space of system (10)–(11) is two dimensional with (*φ*, *σ*) ∈ *S*^1^ × [0, *σ*_max_]. The nullclines are given by *G*(*φ*) = 0 for $\dot{\sigma }=0$ and *σ* = *ω*/sin(*φ* + *α*) for $\dot{\varphi }=0$ in the internal points of the phase space. For the parameter values as in Fig 11, the *φ*-nullcline corresponds to the two lines *φ* = φ* ≈ 0.23 and *φ* = −φ*, while the *σ*-nullcline to a U-shaped nonlinear curve (grey lines in Fig 11).

There is just one fixed point (φ*, σ* = *ω*/sin φ*) of saddle-type within the region *σ* ∈ (0, *σ*_max_). This point is given by the intersection of the nullclines. Fig 11 shows this fixed point and its stable and unstable separatrixes (black lines). An additional fixed point as well as periodic attractor emerge in system (10)–(11) due to the non-smoothness at the boundaries. More specifically, three situations are observed:

**(I)**: One globally stable fixed point *S* = (0, *σ*_max_) which corresponds to the fusion of the two clusters into one. The coupling *σ* = *σ*_max_ and the phase difference is zero at the fixed point, see Fig 11. All orbits are approaching this stable fixed point with time. This corresponding phase portrait is shown in Fig 11(A).
**(II)**: Coexistence of the stable fixed point *S* = (0, *σ*_max_) and a stable periodic orbit, see Fig 11(B). As in the case (I), the fixed point corresponds to the merging of two clusters. The periodic orbit corresponds to two simultaneously existing clusters. The clusters possess different frequencies and, as a result, the phase difference is not bounded and rotate along the circular direction *φ*. Part of the periodic orbit is located on the boundary *σ* = 0, i.e. vanishing inter-cluster coupling. The coupling *σ*(*t*) increases between −φ* and φ* and decreases otherwise. In fact, one can parameterise the coupling *σ* by the phase *φ* on the periodic attractor. In the case when *σ*(φ*) < σ*, the solution returns to the boundary *σ* = 0, moves along it till the orbit reaches the point (−φ*, 0), and the periodic motion repeats.
**(III)**: When max_*φ*_ *G*(*φ*) < 0 then there exists globally stable periodic solution *φ* = *ωt* + *φ*_0_, *σ* = 0. In such a case, the fixed point on the boundary disappears. Formally, this corresponds to an uncoupling between the clusters. However, in the original HH system, this parameter regime corresponds to complete uncoupling of all oscillators because of the depression of all synapses.

In fact, the parameter boundary between the cases (I) and (II) is determined by the condition *σ*(φ*) = σ*, which can be interpreted geometrically as hitting the point (−φ*, 0) by the stable manifold of the saddle equilibrium point, see Fig 11(C). In this special case, the saddle equilibrium attracts the whole set of points from the phase space that is below the stable manifolds, see white area in Fig 11(C). In case (II), the separation between the basins of attraction of the fixed point and the periodic orbit are given by the saddle equilibrium and its stable manifolds. A sufficient condition for the case (III) is given by *c*_*d*_ ≥ *c*_*p*_ and *τ*_*d*_ ≥ *τ*_*p*_. Under these conditions *G*(*φ*) ≤ 0 for all *φ*.

Summarizing, the case (II) corresponds to the situation when clusters are stable and do not merge into one. For this, initial conditions must belong to the basin of attraction of the periodic solution (Fig 11(B), blue domain). The analysis of the phenomenological model indicates that the cluster case always coexists with stable complete synchronization.

#### Comparison of the model and cluster dynamics in HH network

In order to compare dynamics of the phenomenological model (10)–(11) and the original system (1)–(2), we ran a series of simulations of the HH network for parameter values that allow for a stable two-cluster solution. The phases of the clusters are calculated as $\varphi_{1,2}\left(t\right)=2\pi \frac{t-t_{k}}{t_{k+1}-t_{k}}+2\pi k\;\text{for}\;t\in \left[t_{k},t_{k+1}\right)$, where {*t*_1_, …, *t*_*n*_, …} are spiking times with *t*_*k*_ < *t*_*k*+1_ [47]. Correspondingly, the phase difference is *φ*_*HH*_(*t*) = *φ*_1_(*t*) − *φ*_2_(*t*). The coupling measure *σ*_*HH*_ is given by the mean inter-cluster coupling.

**Fig 12:**
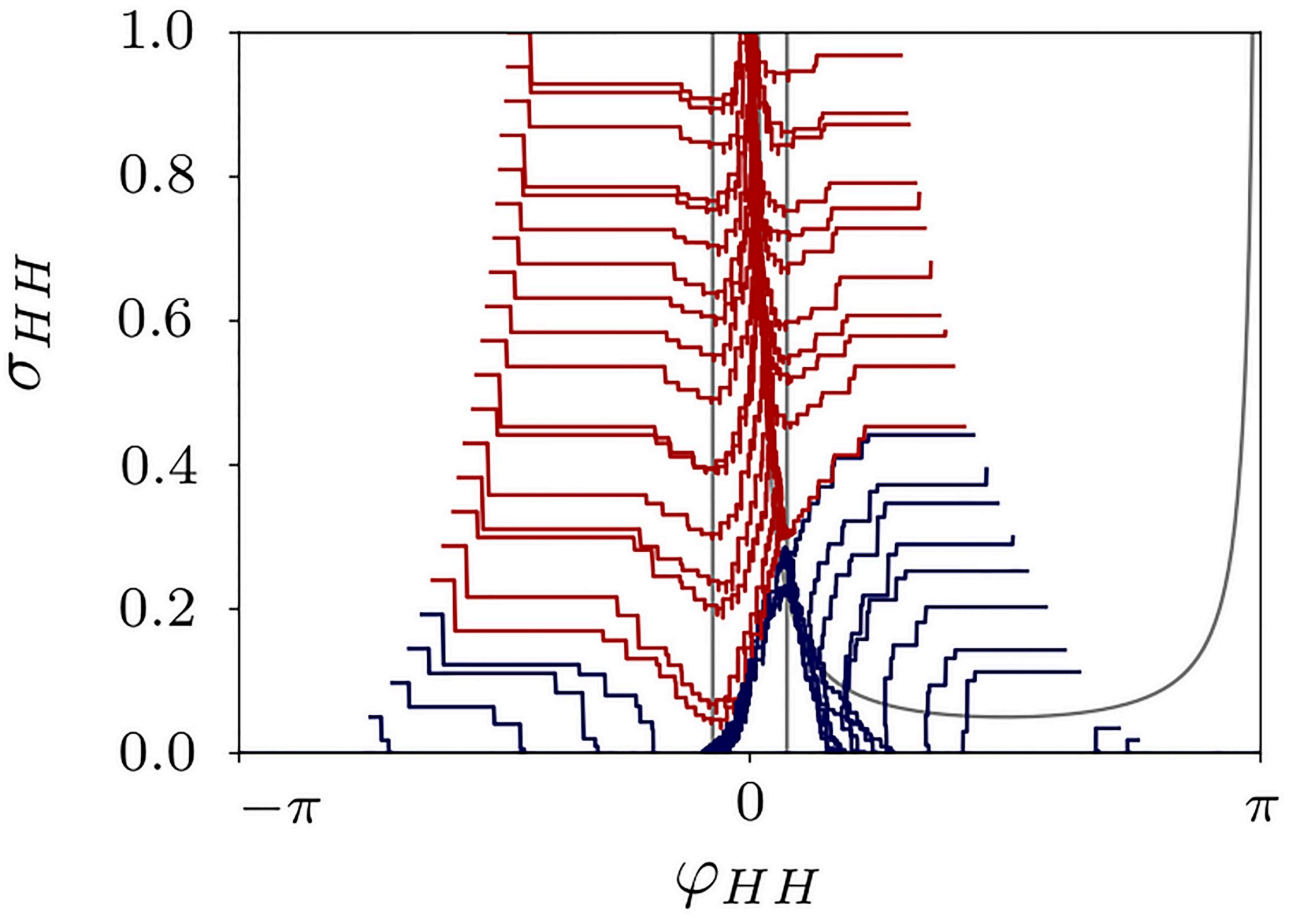
Phase portrait Hodgkin-Huxley model. Dynamics of the phase difference between the clusters *φ*_*HH*_ and mean inter-cluster coupling *σ*_*HH*_ for the solutions of the HH system (1)–(3) for different initial conditions. *N* = 50 with *N*_*s*_ = 7 neurons in the small cluster and *N*_*b*_ = 43 in the big one. Red orbits converge to the regime of complete synchronization, and blue trajectories lead to a stable two-cluster solutions. The nullclines of the phenomenological model are shown in gray. Other parameters: *τ*_*p*_ = 2, *τ*_*d*_ = 5, *c*_*p*_ = 2, *c*_*d*_ = 1.6, and *κ*_max_ = 1.5.

Extracting the quantities *σ*_*HH*_ and *φ*_*HH*_ from the numerically computed solutions of HH system (1)–(3) we obtain a two-dimensional projection of the solution to the plane (*φ*_*HH*_, *σ*_*HH*_), see Fig 12. The discontinuities in the orbits are related to the discrete STDP updates. Additionally, since the phases *φ*_1,2_(*t*) can be firstly accessed after the both clusters fired, some of the area of the phase diagram (see white area in Fig 12) was not accessible. This “empty” area corresponds to anti-phase initial conditions, which are very sensitive, and, after each cluster fires, they appear immediately either in the red or blue area. Nevertheless, the behavior has the same qualitative features as in the phenomenological model, compare Figs 11 and 12.

#### Criteria for the emergence of clusters

Model (10)–(11) can be used to describe plasticity functions, which lead to multiple clusters. For this, we investigate numerically the condition *σ*(φ*) = σ*. More specifically, system (10)–(11) was initialized at the point (−φ*, 0) and numerically integrated forward in time. If *σ*(φ*) < σ*, the two clusters are stable and do not merge. This procedure can be repeated for different parameter values.

**Fig 13:**
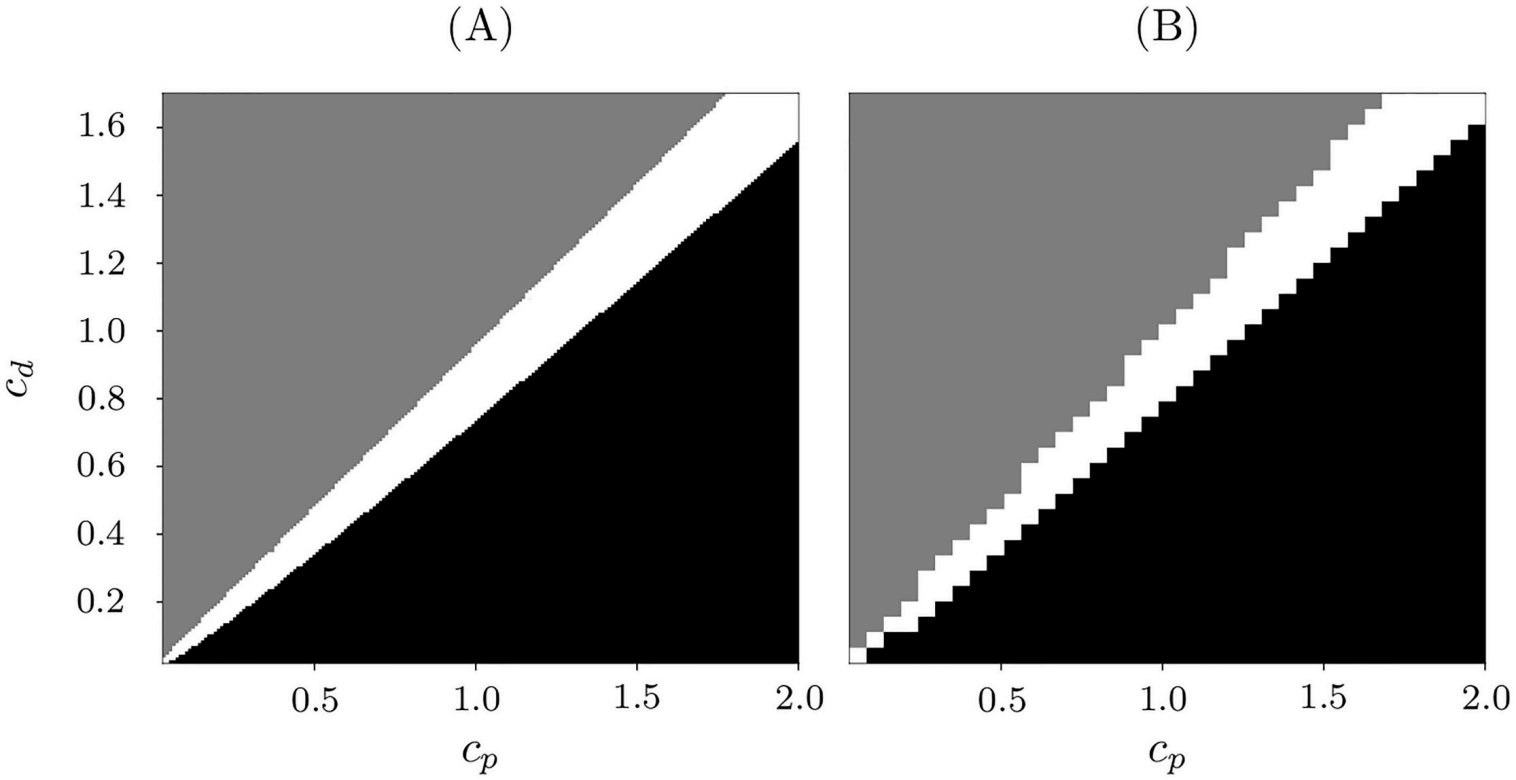
Parameter (*c*_*p*_, *c*_*d*_)-plane of the plasticity function. Panel (A): system (10)–(11). White region: stable periodic solution coexisting with a stable fixed point, case II. Black region: globally stable fixed point, case I. Grey region: globally stable periodic solution with *σ* = 0. Panel (B): original system (1)–(3). White: stable two-clusters (white); black: stable synchrony and no stable clusters; grey: decoupling of all neurons. Other parameters *τ*_*p*_ = 2, *τ*_*d*_ = 5, *N* = 50, *N*_*s*_ = 7, and *κ*_max_ = 1.

In order to restrict the set of plasticity parameters, we fix *τ*_*p*_ = 2 and *τ*_*d*_ = 5 and vary *c*_*p*_ and *c*_*d*_. The results of the simulation are shown in Fig 13(A). The white, black and grey parameter areas correspond to the appearance of stable periodic solution of (10)–(11) (case (II)), globally stable fixed point (case (I)) and the case (III), respectively.

In order to compare the parameter regions obtained for the phenomenological model (Fig 13(A)) with those for the original HH system, we ran numerical simulations of system (1)–(3) with *N* = 50 neurons and *N*_*s*_ = 7 neurons in the small cluster. Starting from the two-cluster state, we monitor the dynamics of the clusters. Fig 13(B) shows the results: the white region corresponds to the case when the clusters survive and stay apart after the simulation time 3000 ms, black—when the clusters merge into one synchronous group, and grey—when the clusters split into uncoupled neurons. This behaviour stays qualitatively the same for different cluster sizes. However, depending on the frequency difference between the clusters, the set of parameters allowing stable cluster states may change its size.

Comparison of the results for the phenomenological system and the HH system in the Fig 13(A) and 13(B) shows that the phenomenological model provides a reasonable approximation.

## Conclusion

Our results show that adaptive neural networks are able to generate self-consistently dynamics with different frequency bands. In our case, each cluster corresponds to a strongly connected component with a fixed frequency. Due to a sufficiently large difference of the cluster sizes and frequencies, the inter-cluster interactions are depreciated, while the intra-cluster interactions are potentiated. In this study, we describe the mechanisms behind the formation and stabilization of these clusters. In particular, we explain why the significant difference between the cluster sizes is important for the decoupling of the clusters. From a larger perspective, the decoupling of the clusters in our case is analogous to the decoupling of timescales in systems with multiple timescales.

Furthermore, we present a two-dimensional phenomenological model which allows for a detailed study of the clustering mechanisms. Despite of the approximations made by the derivation, the model coincides surprisingly well with the adaptive Hodgkin- Huxley network. Using the phenomenological model, we find parameter regions of the plasticity function, where stable frequency clustering can be observed.

Clustering behavior also emerges at the brain scale, where synchronized communities of brain regions constituting large distributed functional networks can intermittently be formed and dissolved [52, 53]. Such clustering dynamics can shape the structured spontaneous brain activity at rest as measured by fMRI. In this study, we show that slow oscillations based on the modulation of synchronized neural activity can already be formed at the resolution level of a single neural population if adaptive synapses are taken into account. These modulations of the amplitude of the mean field can be generated in a stable manner [Fig 9], see also Ref. [35]. The mechanism relies on fluctuations of the extent of synchronization of tonically firing neurons. This is caused by the splitting of the neural population into clusters and the corresponding cluster dynamics. It might contribute to the emergence of slow brain rhythms of electrical (LFP, EEG) and metabolic (BOLD) brain activity reported by [38–41].

However, other mechanisms for generating slow oscillations are possible. The papers [54, 55] discussed the emergence of slow oscillatory activity (< 1Hz) that can be observed in vivo in the cortex during slow-wave sleep, under anesthesia or in vitro in neural populations. The suggested mechanism relies on the corresponding modulation of the firing of individual neurons, and the slow oscillation at the population level was proposed to be the result of very slow bursting of individual neurons that synchronize across the neural population. In contrast, the present work shows that the slow oscillations of the population mean field can also emerge when the firing of individual neurons is not affected. The neurons may tonically fire at high frequencies. The amplitude of the population mean field then oscillates at much lower frequencies due to the slow modulation caused by the cluster dynamics.

Additionally, we would like to mention that the observed frequency clustering resembles phenomenologically the weak chimera states [56, 57] where clusters with different frequencies are formed in symmetrically coupled oscillators without adaptation. However the properties and mechanisms of the appearance of such clusters are different from those presented here, which are essentially based on the slow adaptation.

To conclude, we observe self-organised emergence of clusters in neural networks with STDP. The clustering splits the neural population into groups synchronised at different frequencies, which determine the dynamics of the clusters. These cluster dynamics might play a role in low frequency oscillations during the resting state and can be described by a two-dimensional model.
